# Central retinal artery occlusion following laser treatment for ocular ischemic aortic arch syndrome

**DOI:** 10.3205/oc000036

**Published:** 2015-12-02

**Authors:** Payal J. Shah, Brian Ellis, Lauren R. DiGiovine, Jeffery P. Hogg, Monique J. Leys

**Affiliations:** 1 Riverside Methodist Hospital, Columbus, OH, USA; 2 WVU Eye Institute, Morgantown, WV, USA; 3 Regional Eye Associates, Morgantown, WV, USA; 4 West Virginia University Hospital, Morgantown, WV, USA

**Keywords:** ocular ischemic syndrome, panretinal laser, retrobulbar block, aortic arch, carotid artery stenosis, MRI, carotid duplex ultrasound

## Abstract

**Objective:** Ocular ischemic syndrome is a rare blinding condition generally caused by disease of the carotid artery. We describe a 69-year-old female with a 50 pack-year smoking history with aortic arch syndrome causing bilateral ocular ischemic syndrome.

**Methods:** The patient presented with progressive visual loss and temple pain. Slit lamp biomicroscopy revealed bilateral iris neovascularization. This finding prompted a cardiovascular work up. Panretinal photocoagulation with retrobulbar block was performed in the right eye.

**Results:** A temporal artery biopsy was negative. The carotid duplex ultrasound showed only a 1–39% stenosis. MRA revealed a more proximal occlusion of the aortic branch for which she underwent subclavian carotid bypass surgery. At the one month follow up, the right eye suffered profound vision loss secondary to a central retinal artery occlusion.

**Conclusion:** Ocular neovascularization may be one of the clinical manifestations of aortic arch syndrome. This case also illustrates the limitations of relying solely on carotid duplex ultrasound testing. We caution against overly aggressive panretinal photocoagulation utilizing retrobulbar anesthesia.

## Introduction

Ocular ischemic syndrome (OIS) is a rare condition most commonly associated with decreased ocular perfusion from atherosclerotic disease of either the common or internal carotid artery (ICA) [1], [2]. Carotid duplex ultrasound is often used as the primary means for diagnosis as this imaging modality has a sensitivity of 89% and a specificity of 84% for detecting “high-grade symptomatic carotid artery stenosis” and a sensitivity of 96% and a specificity of 100% for detecting occlusion [1]. However, cases of OIS are missed using a carotid duplex ultrasound due to limitations of the tool for identifying more proximal disease, a high bifurcation of the common carotid artery, or limitations dependent on the operator [1], [2]. In cases suspicious of a false-negative reading on carotid duplex ultrasound, further imaging modalities should be used if the patient’s clinical presentation suggests OIS in order to expedite correct diagnosis and treatment. 

## Case description

**Figure 1:**
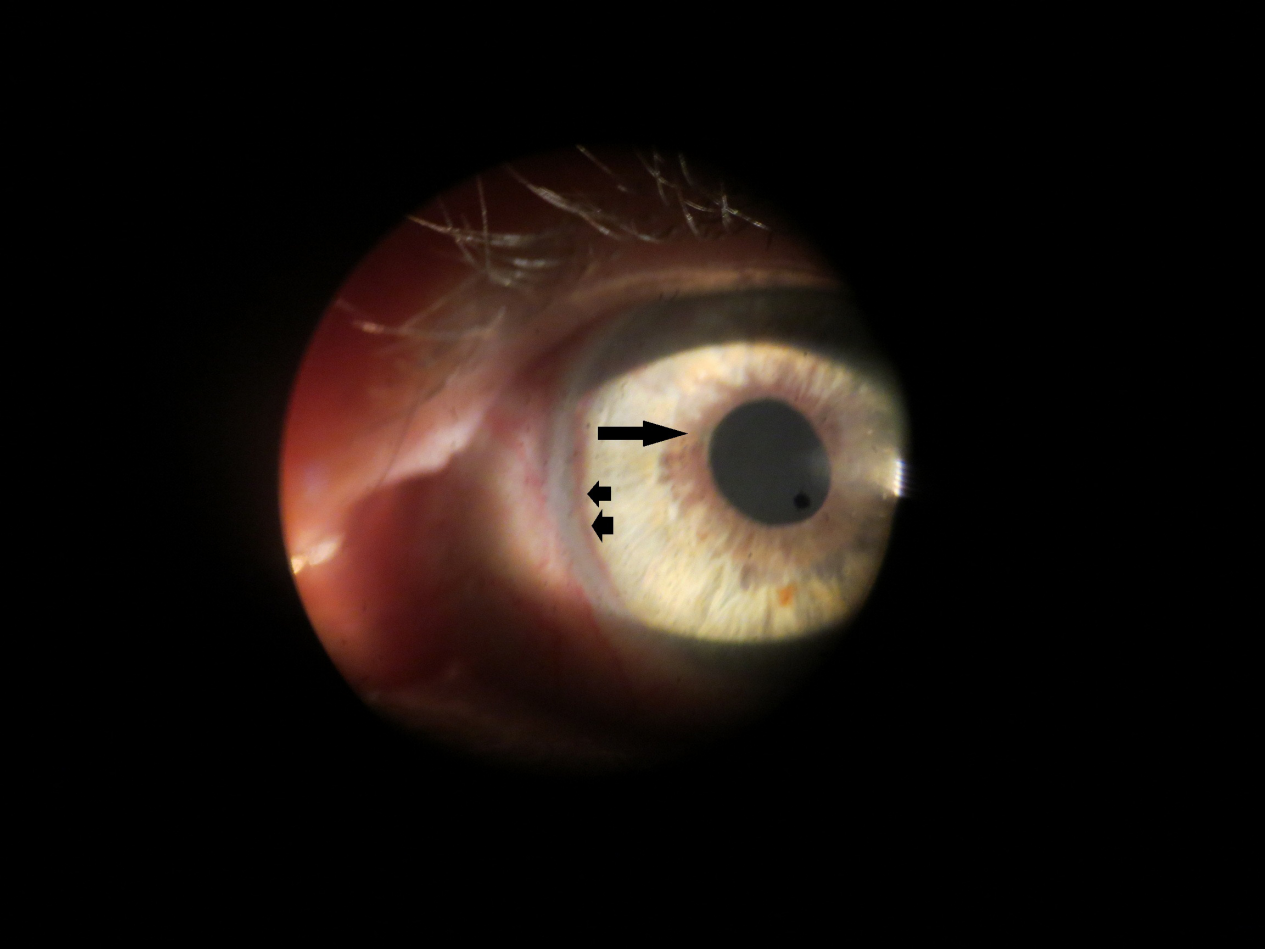
Slit lamp biomicroscopy of the anterior segment of the left eye shows marginal (arrow) and peripheral (arrowheads) circumferential neovascularization of the iris.

**Figure 2:**
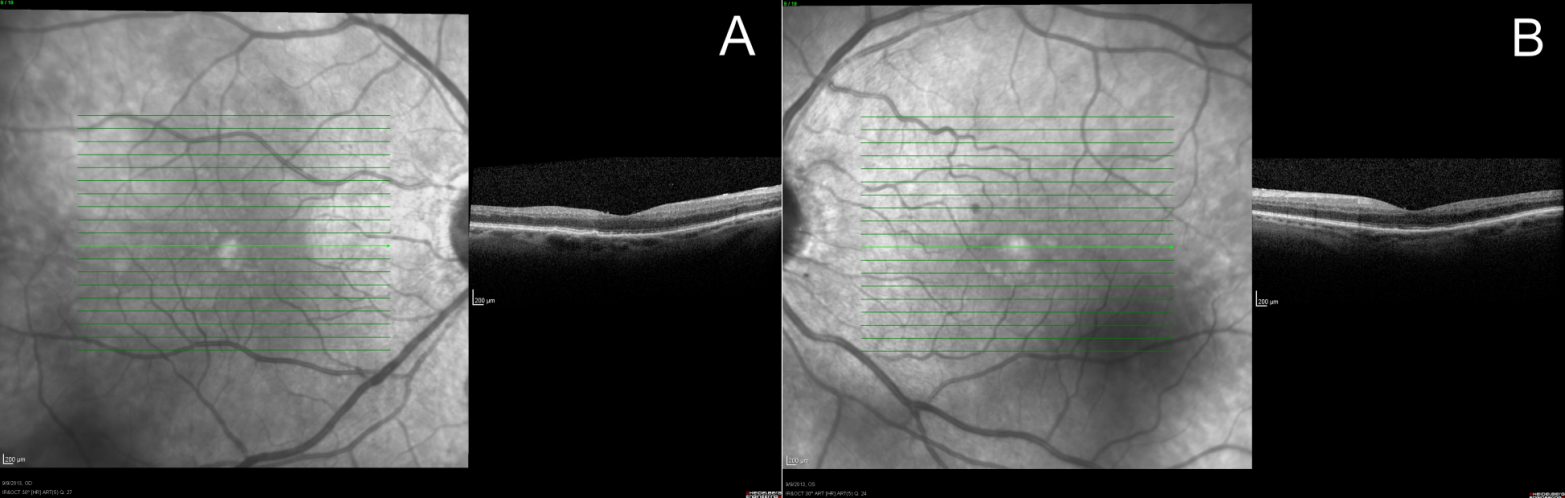
Optical coherence tomography of the right eye (A) and the left eye (B) without evidence of macular edema

A 69-year-old Caucasian female with coronary artery disease, hyperlipidemia, bilateral cataract extraction three years prior, and a fifty pack-year smoking history presented to the clinic with chronic, progressive vision loss greater in the right eye, bilateral photophobia and flashes, and right eye and temple pain. Best corrected visual acuity was 20/50 in both eyes. Pupils were equal and without afferent pupillary defect (APD). Intraocular pressure was 12 mmHg and 10 mmHg in the right and left eyes, respectively. Confrontation visual fields revealed an inferonasal depression in the right eye. Slit lamp biomicroscopy showed iris neovascularization of both eyes (Figure 1 (Fig. 1)). Dilated fundus examination showed unremarkable optic nerves, attenuated arteries, and dilated, non-tortuous veins in both eyes with few drusen in the right macula and very few hemorrhages. Optical coherence tomography showed no evidence of macular edema (Figure 2 (Fig. 2)). 

**Figure 3:**
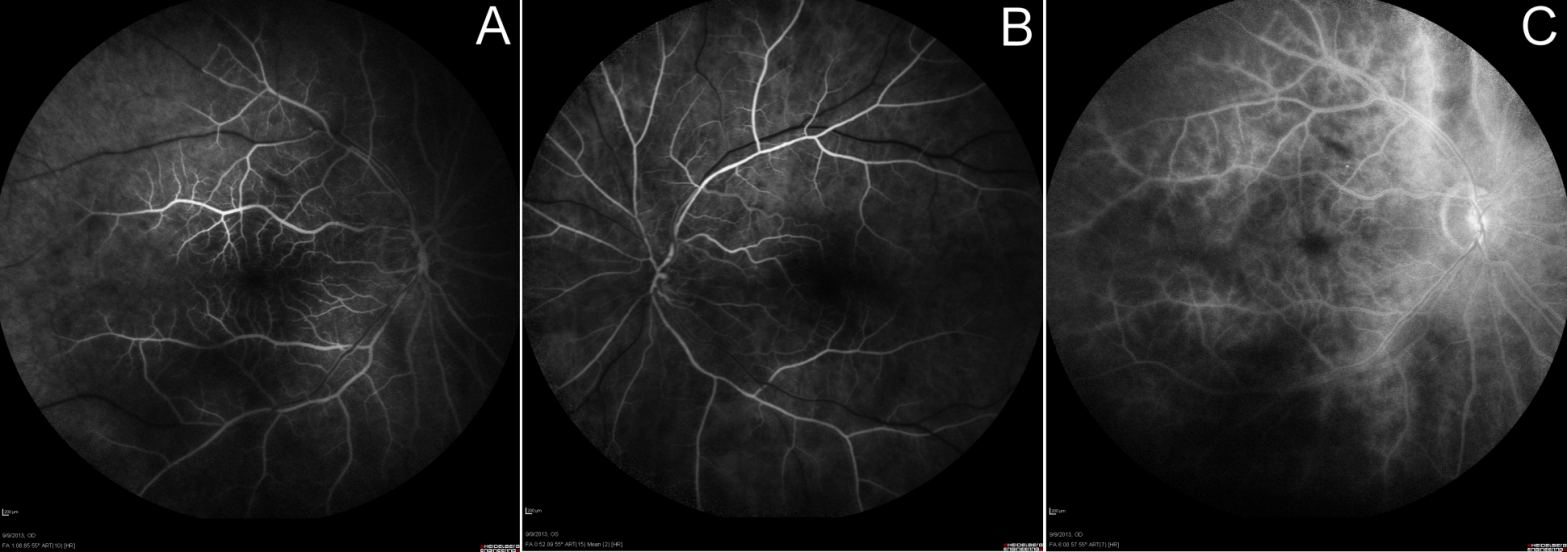
Fluorescein angiogram showing significantly delayed arterial filling with 60 seconds in the right eye (A) and 52 seconds in the left eye (B) and poor peripheral perfusion. Arteries are attenuated and veins are dilated and non-tortuous in both eyes. The late film (C) shows mild capillary leakage in the right eye at 6 minutes but no macular edema or neovascularization.

Fluorescein angiography exhibited delayed arterial filling and poor peripheral perfusion (Figure 3 (Fig. 3)). Right temporal artery biopsy was negative for giant cell arteritis. We ruled out hyperviscosity syndromes, blood dyscrasia, diabetes, Takayasu, collagen vascular disease, thyroid orbitopathy and various infectious causes of retinal ischemia and aortitis [1]. CBC, SPEP, HbA1c, ESR, CRP, FTA-ABS, and hypercoagulation panel were unremarkable. Carotid duplex ultrasound indicated only mild carotid stenosis (1–39%) bilaterally. At the time of initial presentation, the patient was taking Lipitor, Aspirin 81 mg, Relafen, Klonopin, Zoloft, and Nexium. 

**Figure 4:**
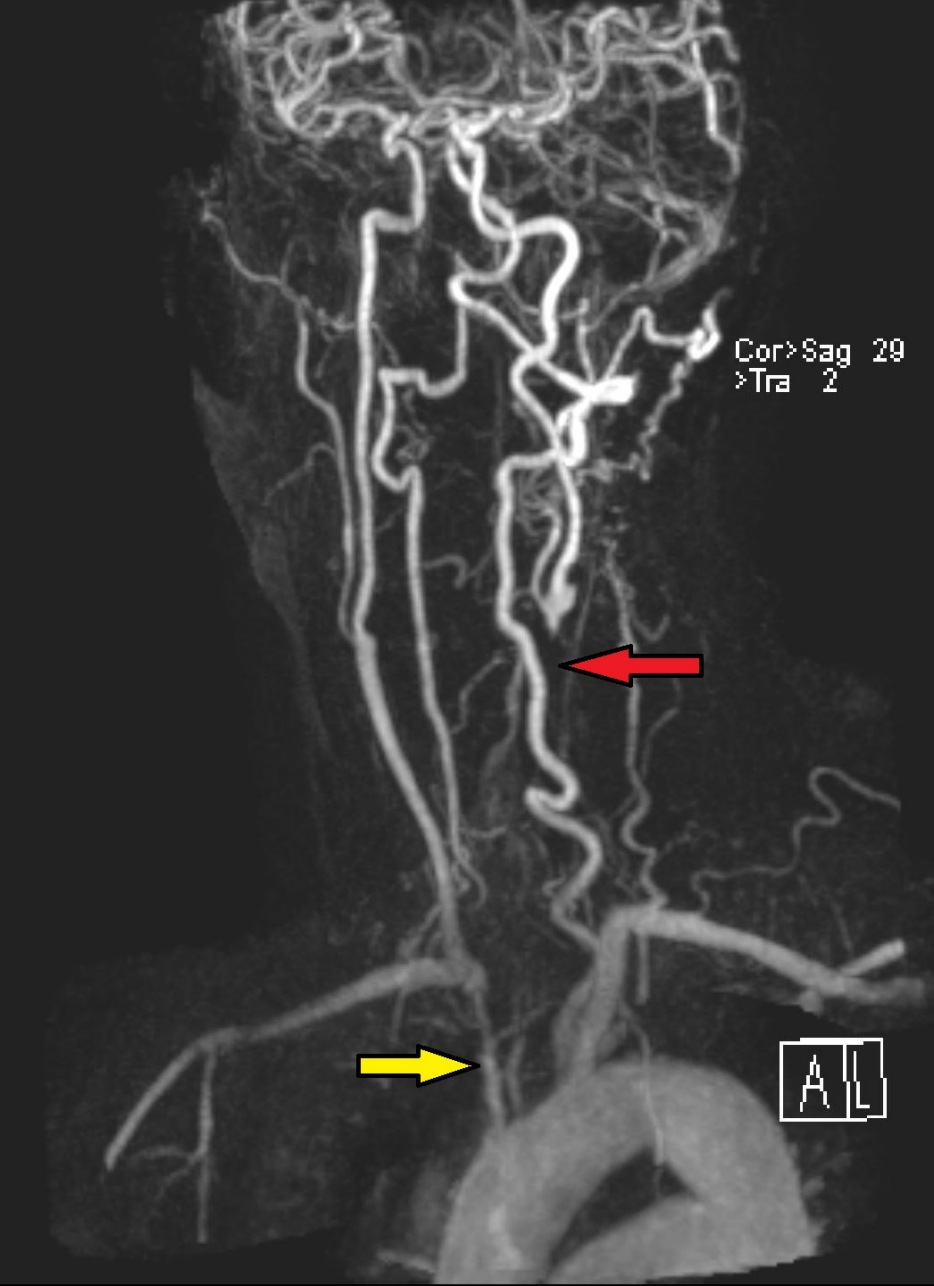
MRA extracranial showing severe narrowing of the origin of the right brachiocephalic artery (yellow arrow) and complete occlusion of the left common carotid artery with distal reconstitution of flow near the bifurcation (red arrow).

The patient received pan-retinal photocoagulation (PRP) in the right eye. Due to low tolerance, a retrobulbar block without epinephrine was administered to the right eye prior to the second PRP ten days later at which time 3,625 spots with a duration of 20 milliseconds of 500 mW were delivered using the indirect laser ophthalmoscope. Eighteen days after this laser session visual acuity had dropped significantly in the right eye to count fingers at 3’ with APD and attenuated posterior vasculature consistent with central retinal artery occlusion. MRA Extracranial showed proximal occlusion of aortic arch branches (Figure 4 (Fig. 4)). Due to concern for diminished blood supply from the aortic arch, the patient received a left subclavian artery to right common carotid artery bypass graft. One month after surgery, neovascular glaucoma developed in the right eye with intraocular pressure of 34 mmHg and 22 mmHg in the left eye. Pressures remained stable on Combigan twice daily. We treated the left eye with short sessions of laser for a total of 2,200 burns (0.05–0.07 sec, 300 micron) using the Laser indirect system for one session and the Varia multicolor slit-lamp system for the remaining 5 sessions. Six months after the bypass surgery, she maintained a visual acuity of 20/50 in the left eye and intraocular pressure was 18 mmHg.

## Discussion

OIS is usually secondary to severe carotid artery stenosis or occlusion which leads to hypoperfusion of the ophthalmic artery, the first branch of the ICA, and subsequent signs of ocular ischemia such as corneal edema, afferent pupillary defect, iris neovascularization, venous dilatation, and mid-peripheral retinal hemorrhages [1], [3], [4]. In fact, OIS patients tend to have greater than 90% stenosis of the common carotid artery or ipsilateral ICA with half of the patients presenting with a complete obstruction of the affected artery [1]. In turn, some consider OIS to be predictive of the severity of carotid stenosis [5]. 

Carotid duplex ultrasound is usually the first-line imaging study used to help diagnosis OIS [1]. However, cases of OIS can be missed due to limitations including inadequate visualization of the common carotid bifurcation due to high bifurcation and less severe ICA disease, and the ultrasound is both operator- and machine-dependent [1]. Therefore, if a patient's clinical presentation suggests OIS but significant stenosis is absent on carotid duplex ultrasound, other imaging studies such as magnetic resonance angiography (MRA) or computed tomographic angiography (CTA) should be used as a second-line tool [1]. Studies show that MRA and CTA are both accurate in detecting severe carotid disease due to stenosis or occlusion [1]. In addition, contrast-enhanced MRA and CTA allow imaging from the aortic arch up the circle of Willis [1]. Use of these imaging modalities can identify more proximal disease, as seen in our patient, and allow for earlier diagnosis and treatment such as vascular surgery.

Carotid reconstructive surgery is known to benefit patients presenting with OIS as they usually suffer from a more serious disease of the carotid arteries. Furthermore, the ophthalmic condition may improve after surgery. A study conducted by Neroev et al. looked at 180 patients with OIS and their visual outcomes after carotid reconstructive surgery. The study determined that carotid artery surgery is beneficial for OIS when done early, prior to the presence of irreversible retinal ischemia or irreversible neovascular glaucoma (NVG) [2]. Though our patient did eventually undergo surgery, the MRA showing proximal disease was not initially obtained as carotid duplex ultrasound showed only mild stenosis. This may have been prevented by recognizing the disagreement between the carotid duplex and the patient’s symptoms and further investigating less common etiologies of OIS such as aortic arch syndrome. 

Aortic arch syndrome, a group of disorders comprised of subclavian steal syndrome, carotid artery occlusion syndrome, and Takayasu arteritis, causes progressive occlusion of branches stemming from the aortic arch [1]. Patients have a more proximal occlusion of the arteries involved that may not be visualized on carotid duplex ultrasound. Symptoms include blurred or loss of vision, transient ischemic attacks or dizziness, and arm weakness or numbness due to ocular, cerebral, or upper extremity hypoperfusion, respectively [1]. Proximal occlusion of branches of the aortic arch can translate into OIS. 

Along with vascular surgery, patients with OIS may benefit from PRP as this procedure can inhibit neovascularization, improve neovascularization of the iris, and prevent NVG which can lead to rapid damage of the optic disc and permanent blindness [3], [6]. Performing a PRP on patients that cannot tolerate pain can be risky as these patients may move their globe frequently during treatment. Ophthalmologists may administer a retrobulbar block which limits ocular motility and sensation, decreasing pain. Though retrobulbar blocks are relatively safe and effective, they are associated with rare complications including: retrobulbar hemorrhage, optic nerve damage and permanent vision loss, central retinal vein occlusion, and central retinal artery occlusion (CRAO) [2], [4], [7]. In our patient, CRAO may have occurred due to retrobulbar block for PRP and orbital edema, vasospasm, and hypoperfusion. Thus, a more cautious approach was taken for treatment in the left eye, and the block was not administered. 

## Conclusions

OIS is a rare but serious condition in which early diagnosis is imperative not only to salvage vision but to promptly treat a more severe underlying diagnosis. With a mortality rate as high as 40% within the first five years of onset, OIS demands a high index of suspicion in patients with multiple risk factors for atherosclerotic disease and ocular signs and symptoms congruent with OIS such as low to normal intraocular pressures, iris neovascularization, dilated non-tortuous retinal veins, and delayed retinal arterial filling, as carotid duplex ultrasound may be falsely negative [1], [3]. Giant cell arteritis should be ruled out as this requires a different treatment approach. Patients should be referred to a cardiologist or vascular surgeon promptly as early reconstructive vascular surgery may improve visual outcomes as well as mortality rates [2], [3]. 

## Notes

### Meeting presentations

The manuscript was presented at the WVU Van Liere research day in Morgantown, WV on April 11, 2014. The manuscript was also presented at the IFan meeting in Paris, France on February 7, 2015. 

### Competing interests

The authors declare that they have no competing interests.
